# Mechanistic study on substitution reaction of a citrato(*p*-cymene)Ru(ii) complex with sulfur-containing amino acids†

**DOI:** 10.1039/c9ra05507j

**Published:** 2019-08-13

**Authors:** Sen-ichi Aizawa, Kohei Takizawa, Momoko Aitani

**Affiliations:** Graduate School of Science and Engineering, University of Toyama 3190 Gofuku Toyama 930-8555 Japan saizawa@eng.u-toyama.ac.jp

## Abstract

The reactions of a dichloro(*p*-cymene)ruthenium(ii) dimer, [RuCl_2_(*p*-cymene)]_2_, with citric acid and sulfur-containing amino acids gave only [Ru(L)(*p*-cymene)]-type complexes (L = citrate (Cit), l-penicillaminate (l-Pen), *S*-methyl-l-cysteinate (*S*-Me-l-Cys) and l-methioninate (l-Met)) in aqueous solutions at various pHs and molar ratios of the reactants, where Cit and the amino acids act as a tridentate ligand. These sulfur-containing amino acid complexes with bound nitrogen, oxygen and sulfur atoms and η^6^-*p*-cymene take *S* absolute configuration around Ru(ii) selectively, having the α-proton oriented in the opposite direction from the Ru(ii) center. The concentration dependences of the observed pseudo-first-order rate constants were provided for the substitution reactions of the citrato complex, [Ru(Cit)(*p*-cymene)], with a large excess of the sulfur-containing amino acids at various temperatures at pH 7.3, where solvolysis path was observed for *S*-Me-l-Cys and l-Met as an intercept but not for l-Pen. The activation parameters for the substitution reactions by the direct attack of the amino acids were changed significantly, indicating that the reaction mechanism varies sensitively with the amino acids from an associative mechanism to an interchange one. The pH dependences of the rate constants of the substitution reactions suggest that the carboxylate group is an attacking group for *S*-Me-l-Cys and l-Met under neutral conditions and the thiol group of l-Pen acts as an entering group constantly at any pH showing a considerably smaller activation energy compared with *S*-Me-l-Cys and l-Met. Differences in stabilities of the amino acid complexes were obtained from the equilibrium constants for the substitution reactions between the amino acids. These results indicate that the activation energies for the substitution reactions of the citrato complex with the amino acids are moderately correlated with the stabilities of the formed amino acid complexes.

## Abstract 2

Thorough kinetic study revealed characteristics of the reaction mechanism for arene ruthenium(ii) complexes with bio-related ligands.

## Graphical abstract

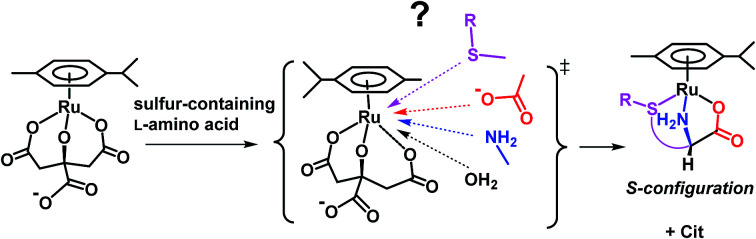

## Introduction

Recently, the catalytic and medicinal properties of arene ruthenium(ii) complexes have been extensively studied. Especially their anticancer activities have received a great deal of attention.^1–9^ On the other hand, arene ruthenium(ii) complexes have been also applied to synthetic studies as a sort of building block for constructing a series of supramolecules.^10,11^ From the pharmacokinetic point of view, thorough studies on the reaction mechanism with bio-related substances in aqueous solution are essential to grasp the chemical species and their transformations *in vivo*. In addition, arene ruthenium(ii) complexes possess a six-coordinate-like electronic feature, while they have a tetrahedral-like three-legged-stool structure. Therefore, elucidation of the reaction mechanism may be worthwhile also from the viewpoint of fundamental coordination chemistry. However, the precise kinetic studies have not been sufficiently carried out so far.

Among second-raw transition metal complexes, ligand substitution reactions of four-coordinate square-planar palladium(ii) complexes have been widely investigated, and an associative mechanism *via* a trigonal–bipyramidal transition state has been generally accepted from the results of kinetic and theoretical studies.^12–17^ These substitution reactions in nucleophilic solvents usually obey a two-term rate law corresponding to a direct attack of the entering ligand and the solvolysis path followed by rapid replacement by the entering ligand.^12^ In this case, the substitution rate depends on donicity or nucleophilicity of the entering ligand and the solvent. On the other hand, if substitution reactions take a dissociative activation mode, bond strength of the leaving ligand becomes the predominant factor to determine the reaction rate. Consequently, elucidation of reaction mechanism is essential for understanding chemical dynamics of metal complexes *in vivo*. In recent years, theoretical calculations have become one of the powerful strategies to propose the activation states. However, kinetic studies are still a straightforward and practical manner to determine the rate law and the effects of solvents, pH and coexisting ions on the reaction rates, which are indispensable to thorough considerations of the reactivity of metal complexes.

While syntheses and properties of some arene ruthenium(ii) complexes with amino acids have been reported so far,^18–27^ it is presently necessary for comprehensible mechanistic studies to adopt simple reaction systems in which one reactant complex gives only one product complex without any by-product under any aqueous conditions such as pH, molar ratio of reactants, coexisting ions, *etc.* in order to keep observation of an identical reaction. Therefore, we started our research from searching metal complexes with one amino acid or organic acid that form only one isomer selectively. As a result, we finally carried out the mechanistic investigation of substitution reactions by using a *p*-cymene ruthenium(ii) complex with citrate as a reactant complex, which is formed *in situ* with *p*-cymene chloro-bridged dimer, [RuCl_2_(*p*-cymene)]_2_. Sulfur-containing amino acids such as *S*-methyl-l-cysteine, l-methionine and l-penicillamine were employed as the entering substrates to give a single product selectively under various conditions, taking advantage of high affinity of thiolate and sulfide sulfur atoms to ruthenium(ii) and the diastereoselective coordination of the potentially tridentate amino acids.

## Experimental section

### Reagents

l-Alanine, l-proline, l-serine, l-threonine, l-aspartic acid, l-glutamic acid, l-methionine, citric acid, [RuCl_2_(*p*-cymene)]_2_ (1) and deuterium oxide (D_2_O) were purchased from Wako Pure Chemical Industries (Osaka, Japan). l-Cysteine, l-penicillamine and sodium 3-(trimethylsilyl)-1-propanesulfonate (DSS) was obtained from Sigma-Aldrich (St. Louis, USA). *S*-Methyl-l-cysteine was purchased from Tokyo Chemical Industry (Tokyo, Japan). l-Valine was obtained from Kanto Chemical Company (Tokyo, Japan). These reagents were used without further purification.

### Measurements

^1^H and ^13^C NMR spectra were recorded on a JNM-A400 FT-NMR spectrometer operating at 399.65 and 100.40 MHz, respectively. In order to determine the chemical shifts, a 3 mm-o.d. NMR tube containing a sample solution was coaxially mounted in a 5 mm-o.d. NMR tube containing deuterated water as a lock solvent and DSS as a reference. Circular dichroism (CD) and UV-vis absorption spectra were recorded on a JASCO J-720WI spectropolarimeter and a PerkinElmer Lambda 950 spectrophotometer, respectively.

## Results and discussion

### Speciation of complexes in solution

**Fig. 1:**
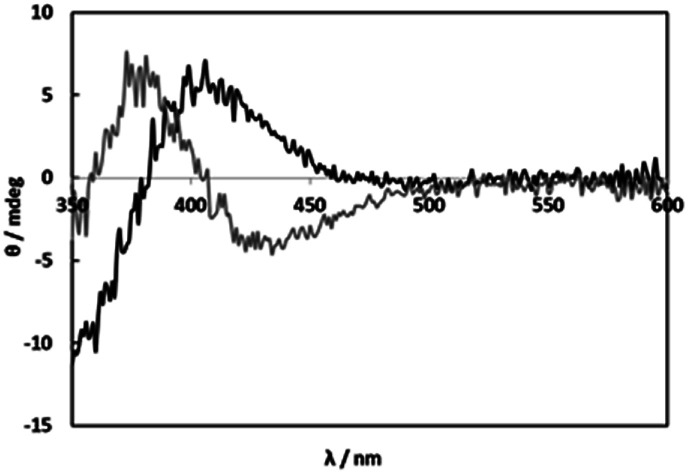
CD spectra of solutions containing 1 (2.00 mM) and l-alanine (4.00 mM) at pH 4.97 (black line) and pH 11.00 (grey line).

The CD spectra of solutions containing [RuCl_2_(*p*-cymene)]_2_ (1) and l-alanine (l-Ala) as a simple amino acid at 1 : 2 molar ratio (1 : 1 molar ratio of Ru(ii) and l-Ala) were measured under weakly acidic (pH 5) and basic (pH 11) conditions (Fig. 1). The totally different CD patterns suggest formations of the carboxylato and amino complexes, respectively. In the weakly acidic solutions at pH 5, the CD spectrum with weak intensity was not changed by an increase in l-Ala concentration (Fig. S1†). This fact shows only the 1 : 1 complex was formed at any concentrations of l-Ala probably due to blocking with chloro ligands. On the other hand, in the basic solutions at pH 11, the weak CD intensity was gradually increased with an increase in l-Ala concentration (Fig. S2†). This indicates that the bis and tris amino complexes were formed successively and the amino coordination is strong enough to be substituted for chloro ligands. The weak CD intensities in the visible region were caused by the vicinal effect of the asymmetric carbon of monodentate l-Ala in the carboxylato and amino complexes.

**Fig. 2:**
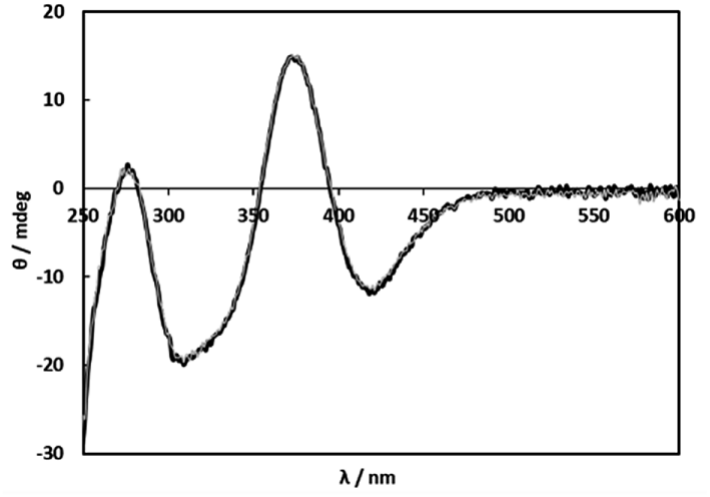
CD spectra of solutions containing 1 (0.200 mM) and *S*-Me-l-Cys (0.400 mM) at pH 2.00 (black line) and pH 11.01 (grey line).

Because selective formation of only one isomer of the 1 : 1 complex under any conditions is suitable for the comprehensible kinetic investigations, nine amino acids, l-proline, l-serine, l-threonine, l-aspartic acid, l-glutamic acid, l-cysteine (l-Cys), *S*-methyl-l-cysteine (*S*-Me-l-Cys), l-methionine (l-Met) and l-penicillamine (l-Pen) besides l-Ala were examined by measuring CD spectra with an increase in molar ratios of the amino acids under acidic and basic conditions. As a result, *S*-Me-l-Cys, l-Met and l-Pen kept the CD spectra unchanged at pH 2 and 11 under a 1 : 1 molar ratio of Ru(ii) and each amino acid (Fig. 2, S3 and S4,† respectively). Furthermore, the CD spectra did not change by an increase in a molar ratio of each amino acid at both pH (Fig. S5 and S6† for *S*-Me-l-Cys, Fig. S7 and S8† for l-Met and Fig. S9 and S10† for l-Pen). It is notable that the CD intensities are one or two orders of magnitude larger than that for monodentate l-Ala (the concentration of Ru(ii) in Fig. 2 was ten times diluted compared with that in Fig. 1).

**Fig. 3:**
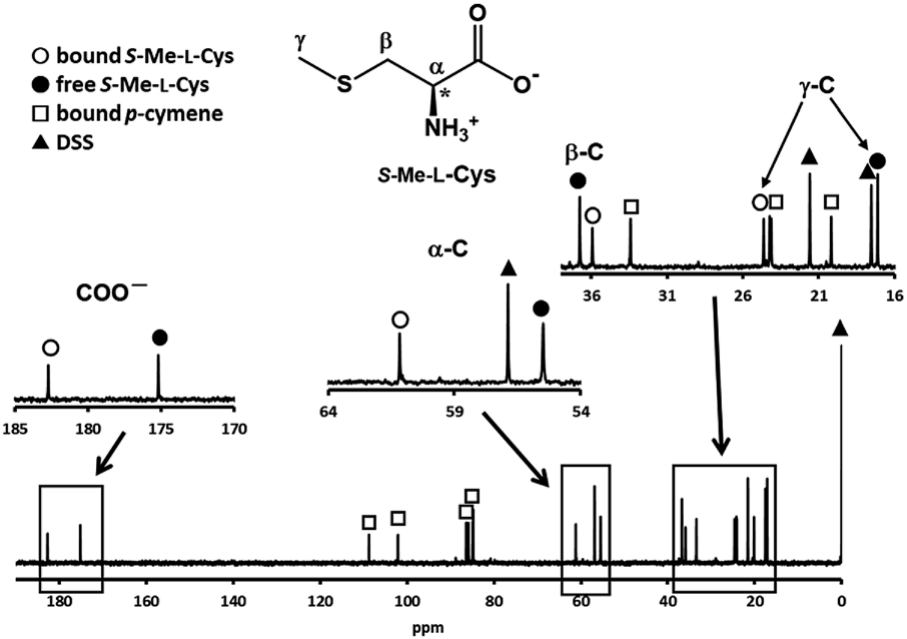
^13^C NMR spectrum of a solution containing 1 and *S*-Me-l-Cys with 1 : 4 molar ratio at pH 2.06 with the signal assignments.

**Fig. 4:**
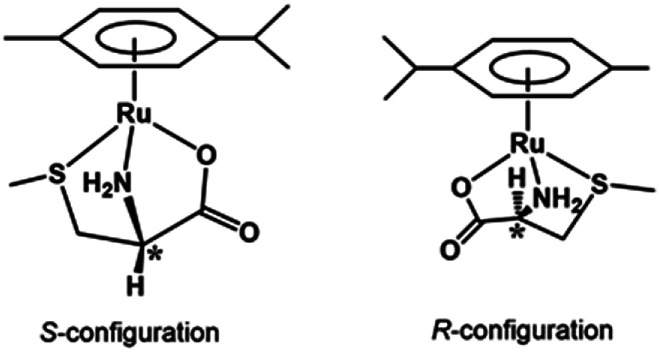
Schematic structures of the *S*-Me-l-Cys complex with *S* and *R* configurations.

Fig. 3 shows the ^13^C NMR spectrum of an acidic solution containing 1 : 4 molar ratio of 1 and *S*-Me-l-Cys (Ru(ii) : *S*-Me-l-Cys = 1 : 2). The signals for free and only one kind of bound *S*-Me-l-Cys and *p*-cymene were observed and considerable down field shifts were shown for the carboxylate, α and γ carbons of the bound *S*-Me-l-Cys indicating formation of only one isomer with coordinated carboxylate, amino and sulfide groups. A little upfield shift of the β carbon may be caused by predominant ring current effect of the bound *p*-cymene ring above it. The ^13^C NMR spectra of solutions containing the l-Met and l-Pen complexes also indicate that these amino acids act as tridentate to form only one isomer of each 1 : 1 complex. The CD patterns with large intensity in the visible region coincide well with one another showing plus and minus CD signs form the longer wavelength side (Fig. S11†). This strongly suggests that these three complexes have the same absolute configuration around Ru(ii). It is probable that *S* configuration is taken around Ru(ii) because *R* configuration is impossible considering the α-proton is forced to be directed to the central metal ion as shown in Fig. 4.

**Fig. 5:**
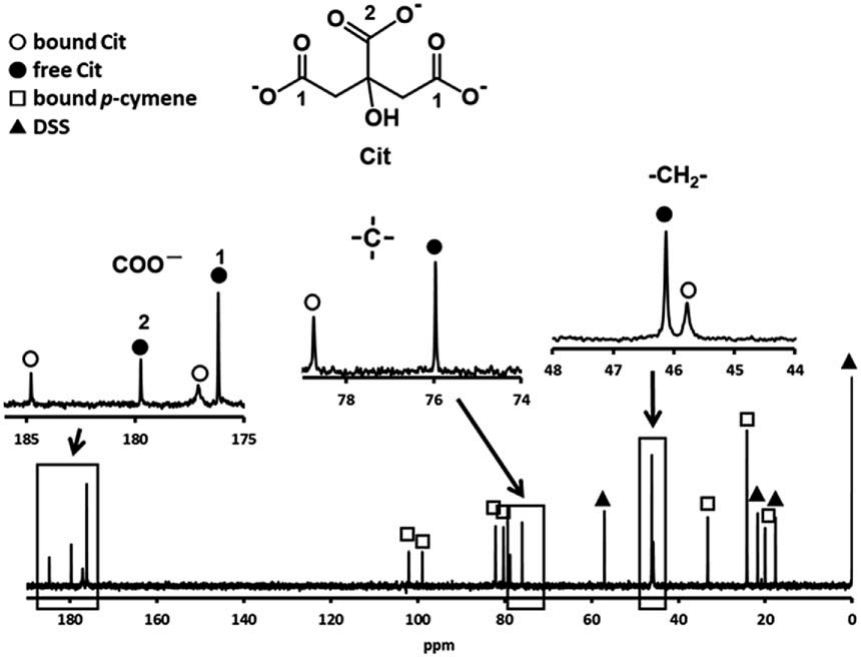
^13^C NMR spectrum of a solution containing 1 and Cit with 1 : 4 molar ratio at pH 3.92 with the signal assignments.

In preliminary experiments, the substitution reactions of the above sulfur-containing amino acid complexes with these amino acids are very slow, then we attempted substitution reactions of a *p*-cymene Ru(ii) complex with a bio-related organic acid, citrate (Cit). Fig. 5 shows the ^13^C NMR spectrum of an acidic solution containing 1 : 4 molar ratio of 1 and Cit (Ru(ii) : Cit = 1 : 2). The signals for free and one kind of bound citrate and *p*-cymene were observed, in which only two signals, small and large ones, were found for the three carboxyl groups of bound citrate. The larger signal at 184.8 ppm with a large downfield shift can be assigned to the equivalently coordinated long-armed two carboxymethyl groups. The large downfield shift of quaternary carbon shows coordination of the deprotonated hydroxyl group. The observed one set of bound Cit and *p*-cymene signals indicates formation of only one isomer with symmetric citrate coordination. It was confirmed from the ^13^C NMR and absorption spectra that the same 1 : 1 complex is formed under pH 4–9.

These results revealed that these sulfur-containing amino acids except for l-Cys and the two six-membered chelate of citrate can make one stable 1 : 1 structure while the other amino acids formed various structures with different denticity and coordinated groups depending on the molar ratio and pH. In the case of l-Cys, some thiolato-bridged structures can be formed^20,28–35^ because there are not any methyl groups adjacent to the thiolate group as in l-Pen, which can give steric hindrance not to form sulfur-bridging structures.

### Kinetics

**Fig. 6:**
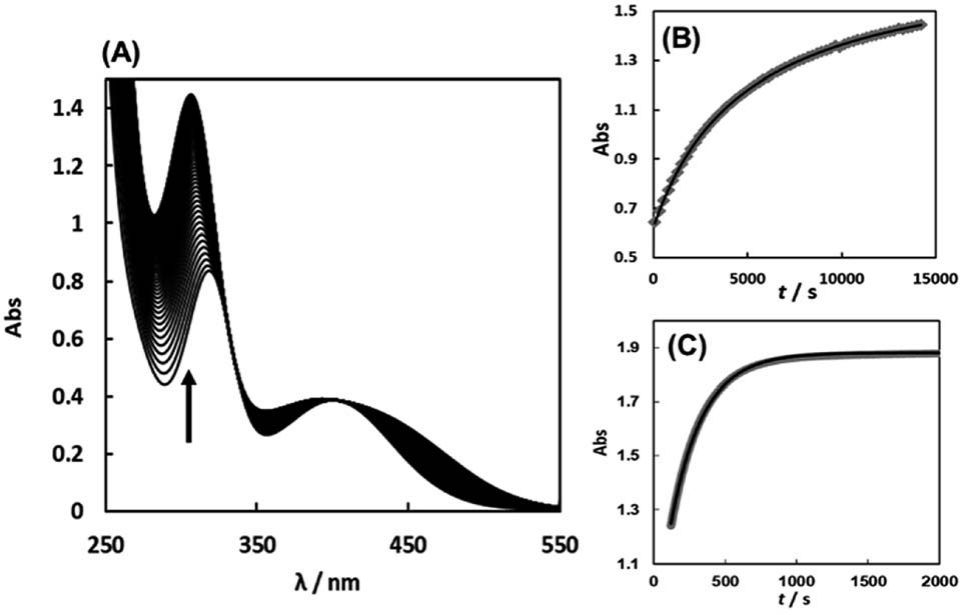
Absorption spectral change for the reaction of the Cit complex (0.80 mM) with 1 eq. of l-pen at pH 7.34 (298.2 K) with the arrow showing the direction of the spectral change (A). The absorbance changes at 305 nm with time (grey square) for the above reaction conditions (B) and for the reaction with 30 eq. of l-pen at pH 7.41 (298.9 K) (C) with calculated curves for the second order reaction (black line in (B)) and for the first order reaction (black line in (C)), respectively.

The substitution reactions of the Cit complex with the sulfur-containing amino acids were carried out at neutral pH similarly *in vivo*. Fig. 6(A) shows the absorption spectral change for the reaction of the Cit complex with 1 eq. of l-Pen. The absorbance change at 305 nm is shown in Fig. 6(B), which was in good agreement with a second-order reaction curve. When a large excess of l-Pen (30 eq.) was used, the absorbance change was completely fitted to a first-order reaction curve (Fig. 6(C)). To carry out the pseudo-first-order reactions with l-Pen, *S*-Me-l-Cys and l-Met, it was confirmed that formations of the 1 : 1 complexes proceed even in the presence of an extremely large excess of these three amino acids. The CD spectra after completion of the reactions at pH 7.2 in the presence of 80–150 eq. of the amino acids were almost the same as those for the 1 : 1 reaction, respectively (Fig. S12–S14†). The absorbance changes for the substitution reactions with a large excess of the three amino acids were in good agreement with first-order reaction curves and the pseudo-first-order observed rate constants (*k*_obs_s) were obtained by using the absorbance changes at 305 nm, 358 nm and 413 nm for l-Pen, *S*-Me-l-Cys and l-Met, respectively.

**Fig. 7:**
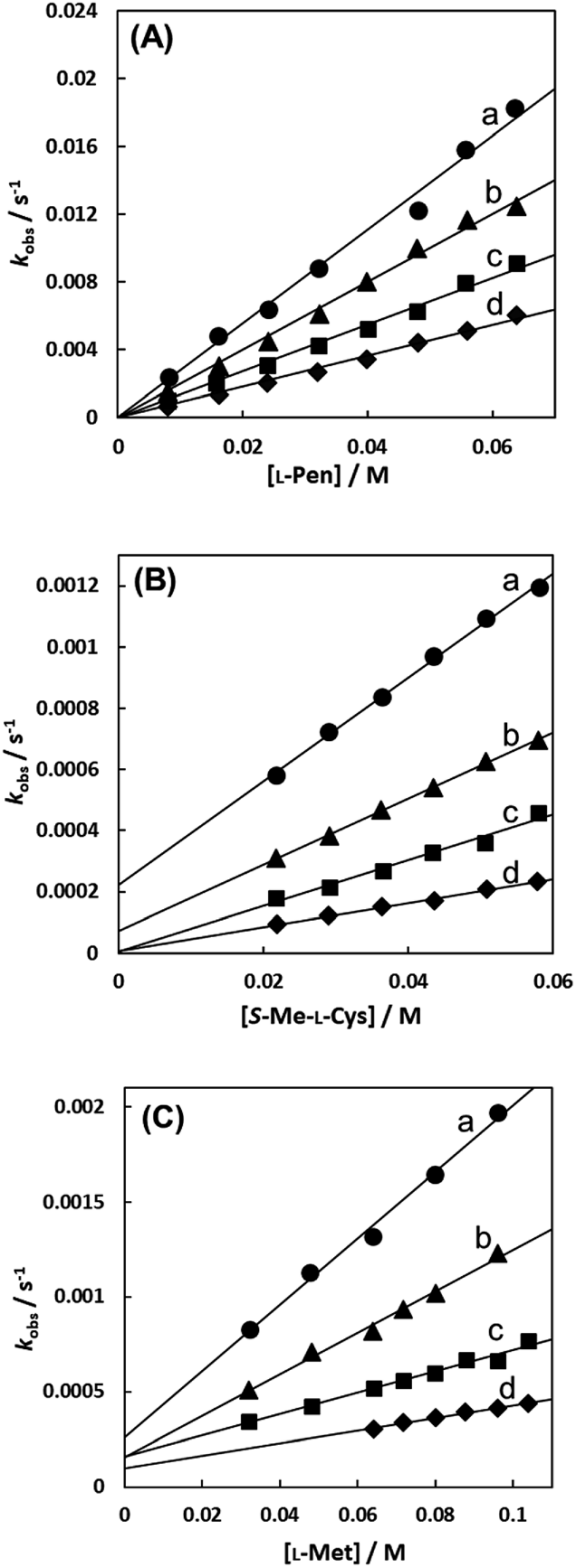
Amino acid concentration dependences of observed rate constants for substitution reactions of the Cit complex (0.80 mM) with l-Pen (A) at 303.8 K (a), 298.9 K (b), 293.9 K (c) and 288.7 K (d), *S*-Me-l-Cys (B) at 309.1 K (a), 303.8 K (b), 298.9 K (c) and 293.9 K (d) and l-Met (C) at 324.2 K (a), 319.3 K (b), 314.2 K (c) and 309.1 K (d) at pH 7.3.

Fig. 7(A)–(C) show the amino acid concentration dependences of *k*_obs_s under the pseudo-first-order conditions at various temperatures at pH 7.3. Apparent intercepts were observed for the reactions with *S*-Me-l-Cys and l-Met. The intercept is usually observed for the reverse reaction or solvolysis. Because the revers reaction must be also second order as observed for the forward reaction, the absorption change should become gradually deviated from first order with generation of free Cit by progress of the substitution reaction unless establishing pseudo-first-order conditions also for the reverse reaction by coexistence of a large excess of Cit. However, the actual absorbance changes were completely consistent with a first-order reaction through to completion of the reaction. Furthermore, the CD spectra of the amino acid complexes were not changed at all by addition of Cit. These facts indicate that no reverse reaction proceeded and remaining possibility is solvolysis.

**Fig. 8:**
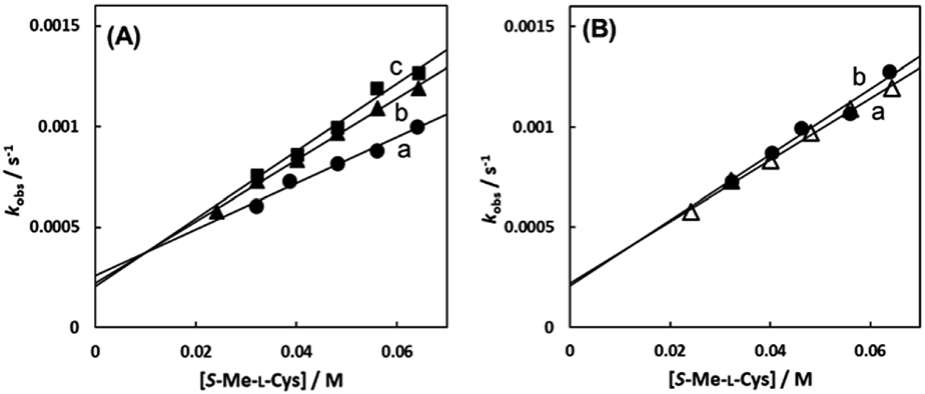
*S*-Me-l-Cys concentration dependences of observed rate constants for substitution reactions of the Cit complex (0.80 mM) at 309.1 K at pH 5.0 (a), 7.0 (b) and 9.0 (c) (A) and for the solution containing 0 M (a) and 0.1 M (b) of NaCl at pH 7.0 (B).

As shown in Fig. 8, the intercept did not depend on pH or chloride concentration. These facts strongly suggest that the intercept is attributed to solvolysis reaction with water, not to hydroxide or dissociated chloride attack or proton-assisted reaction. A decrease in the slope at pH 5 suggests the retardation by protonation of the attacking carboxylate group of the amino acid and a slight increase in the slope at pH 9 may be attributable to participation of the deprotonated amino group as an attacking group.

**Fig. 9:**
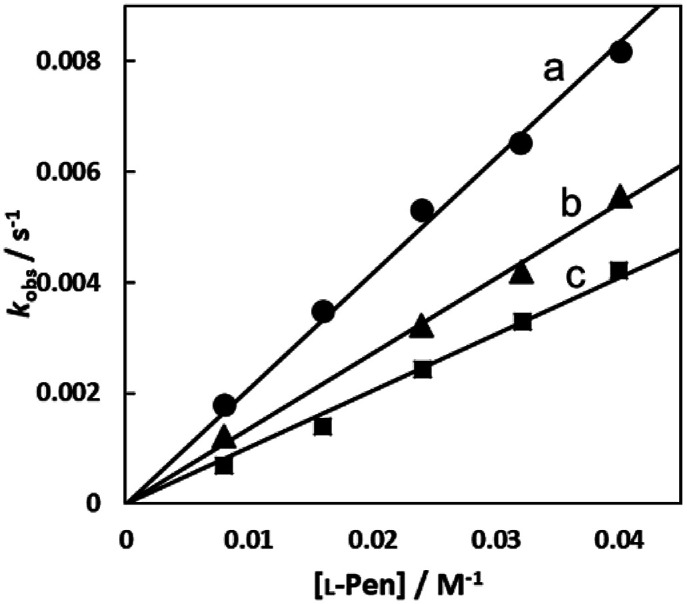
l-Pen concentration dependences of observed rate constants for substitution reactions of the Cit complex (0.80 mM) at pH 7.0 (a), 8.0 (b), and 9.0 (c) (298.2 K).

In the case of the concentration dependence of *k*_obs_ for l-Pen, remarkably large slopes compared with the reactions with *S*-Me-l-Cys and l-Met were observed without an intercept as shown in Fig. 7(A). Furthermore, the slope was decreased with an increase in pH (Fig. 9) contrary to the pH dependences of *k*_obs_s for *S*-Me-l-Cys and l-Met. The especially large second-order rate constants (*k*s) obtained by the slopes suggest that the thiol group instead of carboxylate one is an attacking group, which is not possessed in *S*-Me-l-Cys or l-Met. The retardation with an increase in pH is consistent with the low p*K*_a_ values of l-Pen (1.8, 7.9, 10.7), by which the anionic species with the deprotonated amino group is generated at around pH 8.^36^ The negative charge of the entering ligand gives rise to electrostatic repulsion with the dianionic Cit complex to prevent the entering ligand attack onto the central Ru(ii) ion, though the other two amino acids are uncharged under a neutral condition (p*K*_a_ values: 2.0 and 8.7 for *S*-Me-l-Cys and 2.3 and 9.1 for l-Met).^37^ These facts are also consistent with the assumption that the donating thiol group in l-Pen acts as an entering group constantly at any pH.

**Fig. 10:**
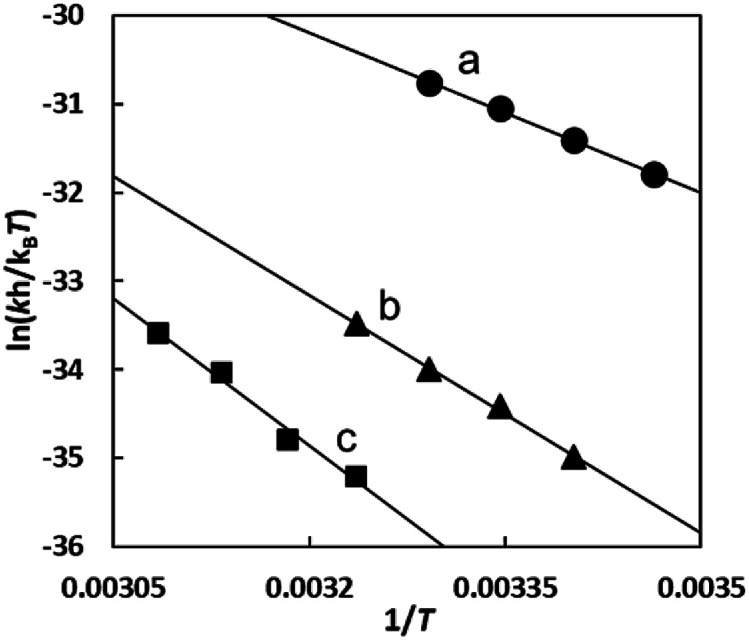
Eyring plots for substitution reactions of the Cit complex with l-Pen (a), *S*-Me-l-Cys (b) and l-Met (c) at pH 7.3.

**Fig. 11:**
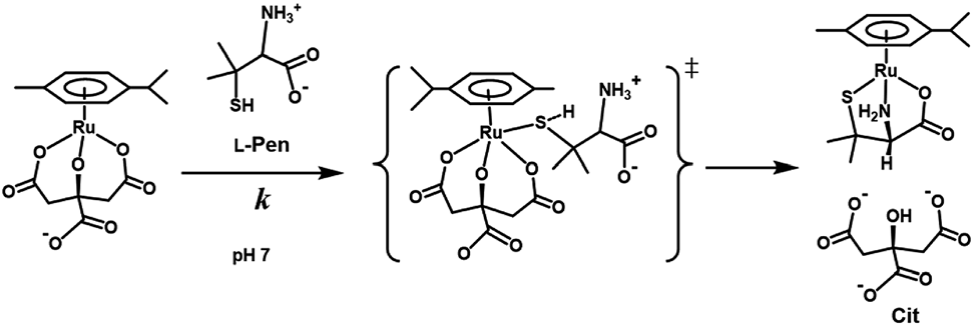
Proposed reaction mechanism of substitution reaction of the Cit complex with l-Pen at pH 7.

**Fig. 12:**
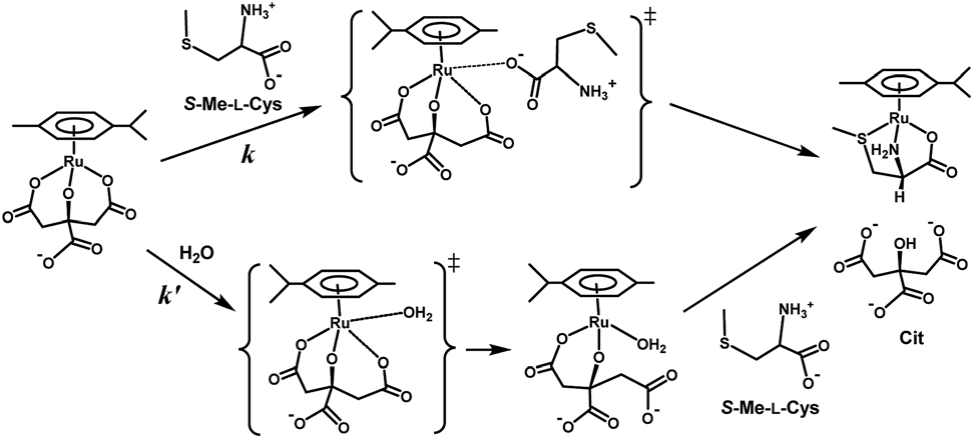
Proposed reaction mechanism of substitution reaction of the Cit complex with *S*-Me-l-Cys at pH 7.

Fig. 10 shows the Eyring plots for the second-order rate constants obtained from the slopes in Fig. 7(A)–(C) and the activation parameters at pH 7.3 are given in Table 1. These results indicate that the reaction mechanism changes significantly with amino acids, that is, from an associative mechanism for l-Pen to a rather interchange one for l-Met. The much smaller activation energy for l-Pen obtained from the rate constant, *k*_298_, suggests that the thiol sulfur attack is effective for the substitution reaction. As shown in Fig. 11, the strong thiol donor attack with concerted proton dissociation can be proposed for the substitution reaction with l-Pen. The low activation energy of this substitution made the solvolysis path negligible. On the other hand, the anionic carboxylate attack under a neutral condition, which reasonably depends on pH, and the competitive solvolysis path can be proposed for *S*-Me-l-Cys (Fig. 12) and l-Met.

**Table 1:** Rate constants at 298 and activation parameters for substitution reactions of the Cit complex with l-Pen, *S*-Me-l-Cys and l-Met at pH 7.3

Amino acid	*k*_298_/M^−1^ s^−1^	Δ*G*^‡^_298_/kJ mol^−1^	Δ*H*^‡^/kJ mol^−1^	Δ*S*^‡^/J mol^−1^ K^−1^
l-Pen,	2.1 × 10^−1^	77.1 ± 0.1	50.2 ± 1.3	−90.5 ± 4.5
*S*-Me-l-Cys	7.5 × 10^−3^	85.6 ± 0.1	74.5 ± 2.2	−37.4 ± 7.3
l-Met	8.9 × 10^−4^	90.7 ± 0.2	92.1 ± 3.4	4.8 ± 11.0

**Fig. 13:**
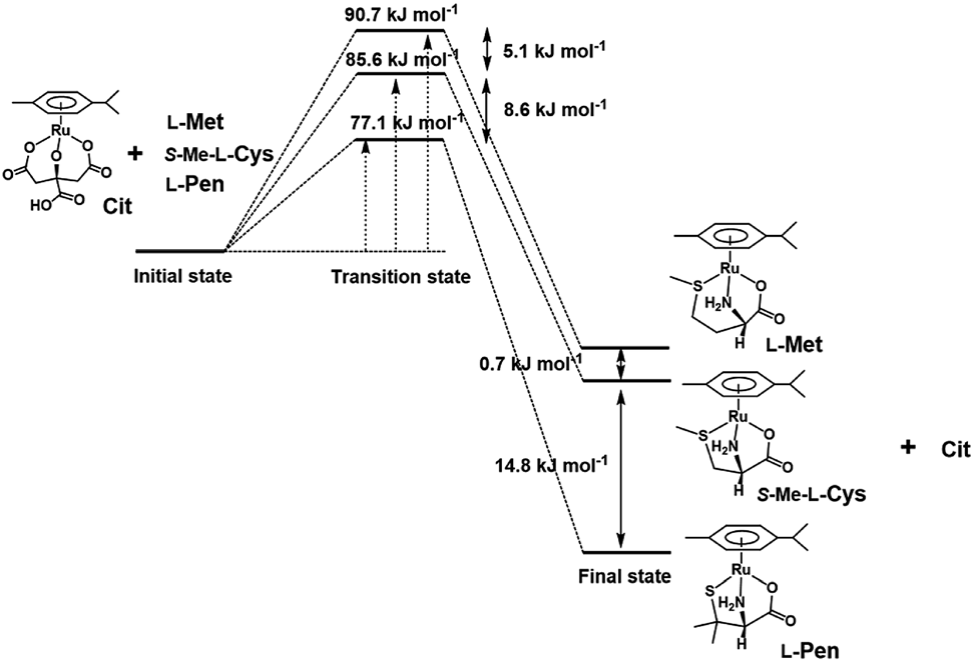
Reaction coordinates of substitution reactions of the Cit complex with l-Pen, *S*-Me-l-Cys and l-Met at pH 7.

To compare stabilities of the amino acid complexes, the equilibrium constants for the substitution reactions of the *S*-Me-l-Cys complex with l-Met and l-Pen at 298 K were obtained at pH 7.3 from the ^1^H NMR signal integration as shown in Table 2 together with Δ*G*^0^s at 298 K. The Δ*G*^0^_298_ value for the substitution reaction of the l-Pen complex with l-Met at pH 7.3 are consistent with that calculated from the above two Δ*G*^0^_298_ values. If the solvation energy of the free amino acids can be regarded as similar under a neutral condition, the reaction coordinates can be depicted as shown in Fig. 13. It is revealed that the thiolato complex with l-Pen is most stabilized among these three complexes and the five-membered *N*,*S*-chelate is a little preferable to the six-membered one by comparison between two methyl sulfide complexes with *S*-Me-l-Cys and l-Met. Furthermore, differences in the activation energies for the substitution reactions are consistent with differences in the stabilities of the formed complexes. This reaction coordinates suggest that the energy levels of the partially formed complexes in the transition state are similarly influenced by the entering ligands as in the final states.

**Table 2:** Equilibrium constants and Δ*G*^0^s for substitution reactions of the *S*-Me-l-Cys complex with l-Pen and l-Met and those of the l-Met complex with l-Met at pH 7.3

Substitution reactions	*K*_298_	Δ*G*^0^_298_/kJ mol^−1^
*S*-Me-l-Cys with l-Met	7.5 × 10^−1^	0.7
*S*-Me-l-Cys with l-Pen	4.0 × 10^2^	−14.8
l-Pen with l-Met	2.0 × 10^−3^	15.2

## Conclusion

In conclusion, the results of this study indicate that the sulfur-containing amino acids such as l-Pen, *S*-Me-l-Cys and l-Met form stable [Ru(L)(*p*-cymene)]-type complexes with *S* configuration around Ru(ii) center, where the order of stability for ligand L is l-Pen > *S*-Me-l-Cys > l-Met. The activation energies for the substitution reactions of [Ru(Cit)(*p*-cymene)]^2−^ with these amino acids under a neutral condition are moderately correlated with the stabilities of the amino acid complexes, and the reaction mechanism changes significantly from an associative mechanism for l-Pen to an interchange one for l-Met. The thiol group of l-Pen is the attacking group for the substitution reaction constantly, while the carboxylate group acts as the attacking group for *S*-Me-l-Cys and l-Met under a neutral condition and the amino group attacking may be also possible at higher pH. Furthermore, solvolysis path was also observed for the reaction with *S*-Me-l-Cys and l-Met. These results shows that the reaction mechanism depends sensitively on the attacking donor groups of amino acids and reaction rate changes with the stabilization caused by the entering amino acids in the transition state.

## Conflicts of interest

There are no conflicts to declare.

## Supplementary Material

RA-009-C9RA05507J-s001

## References

[cit1] Timerbaev A. R., Hartinger C. G., Aleksenko S. S., Keppler B. K. (2006). Chem. Rev..

[cit2] Dyson P. J., Sava G. (2006). Dalton Trans..

[cit3] Fink G. S. (2010). Dalton Trans..

[cit4] Hanif M., Henke H., Meier S. M., Martic S., Labib M., Kandioller W., Jakupec M. A., Arion V. B., Kraatz H.-B., Keppler B. K., Hartinger C. G. (2010). Inorg. Chem..

[cit5] Haghdoost M. M., Guard J., Golbaghi G., Castonguay A. (2018). Inorg. Chem..

[cit6] Rojas O. A. L., Robalo M. P., Tomaz A. I., Carvalho A., Fernandes A. R., Marques F., Folgueira M., Yáñez J., García D. V., Torres M. L., Fernández A., Fernández J. J. (2018). Inorg. Chem..

[cit7] Pettinari R., Marchetti F., Nicola C. D., Pettinari C., Galindo A., Petrelli R., Cappellacci L., Cuccioloni M., Bonfili L., Eleuteri A. M., da Silva M. F. C. G., Pombeiro A. J. L. (2018). Inorg. Chem..

[cit8] Lari M., Alonso M. M., Busto N., Manzano B. R., Rodríguez A. M., Acuña M. I., Domínguez F., Albasanz J. L., Leal J. M., Espino G., García B. (2018). Inorg. Chem..

[cit9] Lam N. Y. S., Truong D., Burmeister H., Babak M. V., Holtkamp H. U., Movassaghi S., Tora D. M. A., Zafar A., Kubanik M., Oehninger L., Söhnel T., Reynisson J., Jamieson S. M. F., Gaiddon C., Ott I., Hartinger C. G. (2018). Inorg. Chem..

[cit10] Severin K. (2006). Chem. Commun..

[cit11] Paul L. E. H., Therrien B., Furrer J. (2012). Inorg. Chem..

[cit12] Wilkins R. G. , Kinetics and Mechanism of Reaction of Transition Metal Complexes, VCH, Weinheim, 2nd edn, 1991, ch. 4

[cit13] Cross R. J. , Mechanisms of Inorganic and Organometallic Reactions, ed. M. V. Twigg, Plenum, New York, 1988, ch. 5

[cit14] Lin Z., Hall M. B. (1991). Inorg. Chem..

[cit15] Tubino M., Merbach A. E. (1983). Inorg. Chim. Acta.

[cit16] Ducommun Y., Helm L., Merbach A. E., Hellquist B., Elding L. I. (1989). Inorg. Chem..

[cit17] Hallinan N., Besancon V., Forster M., Elbaze G., Ducommun Y., Merbach A. E. (1991). Inorg. Chem..

[cit18] Carmona D., Mendoza A., Lahoz F. J., Oro L. A. (1990). J. Organomet. Chem..

[cit19] Severin K., Bergs R., Beck W. (1998). Angew. Chem., Int. Ed..

[cit20] Wang F., Chen H., Parkinson J. A., Murdoch P. S., Sadler P. J. (2002). Inorg. Chem..

[cit21] Wang F., Bella J., Parkinson J. A., Sadler P. J. (2005). J. Biol. Inorg Chem..

[cit22] Lu X. L., Zhang L., Lou J.-D., Yan J., Nong P.-S., Chen X.-H., Yang J.-J., Gao M. (2010). Transition Met. Chem..

[cit23] Paul L. E. H., Therrien B., Furrer J. (2012). J. Biol. Inorg Chem..

[cit24] Bíró L., Balogh E., Buglyó P. (2013). J. Organomet. Chem..

[cit25] Egbewande F. A., Paul L. E. H., Therrien B., Furrer J. (2014). Eur. J. Inorg. Chem..

[cit26] Bihari Z., Nagy Z., Buglyó P. (2015). J. Organomet. Chem..

[cit27] Scrase T. G., O'Neill M. J., Peel A. J., Senior P. W., Matthews P. D., Shi H., Boss S. R., Barker P. D. (2015). Inorg. Chem..

[cit28] Okamoto K., Aizawa S., Konno T., Einaga H., Hidaka J. (1986). Bull. Chem. Soc. Jpn..

[cit29] Aizawa S., Okamoto K., Einaga H., Hidaka J. (1988). Bull. Chem. Soc. Jpn..

[cit30] Konno T., Aizawa S., Okamoto K., Hidaka J. (1990). Bull. Chem. Soc. Jpn..

[cit31] Konno T., Okamoto K. (1995). Bull. Chem. Soc. Jpn..

[cit32] Aizawa S., Sone Y., Khajar S., Ohishi Y., Yamada S., Nakamura M. (2000). Bull. Chem. Soc. Jpn..

[cit33] Aizawa S., Tsubosaka S. (2016). Chirality.

[cit34] Furrer J., Fink G. S. (2016). Coord. Chem. Rev..

[cit35] Stíbal D., Riedel T., Dyson P. J., Fink G. S., Therrien B. (2016). Inorg. Chim. Acta.

[cit36] Sisombath N. S., Jalilehvand F., Schell A. C., Wu Q. (2014). Inorg. Chem..

[cit37] Shehata M. R., Shoukry M. M., Nasr F. M. H., van Eldik R. (2008). Dalton Trans..

