# Supplementary material for: Mechanistic study on substitution reaction of a citrato(p-cymene)Ru(ii) complex with sulfur-containing amino acids
Source: RSC Adv. 2019 Aug 13;9(43):25177–83. doi: 10.1039/c9ra05507j (PMC9069928; doi:10.1039/c9ra05507j)
Supplement: RA-009-C9RA05507J-s001 [file RA-009-C9RA05507J-s001.pdf]

Electronic Supplementary Material (ESI) for RSC Advances.
This journal is © The Royal Society of Chemistry 2019

# Electronic Supporting Information (ESI)

**Mechanistic Study on Substitution Reaction of Citrato(*p*-cymene)Ru(II) Complex with Sulfur-Containing Amino Acids**

Sen-ichi Aizawa,* Kohei Takizawa and Momoko Aitani

*Graduate School of Science and Engineering, University of Toyama, 3190 Gofuku, Toyama 930-8555, Japan, saizawa@eng.u-toyama.ac.jp*

Figs. S1-S14

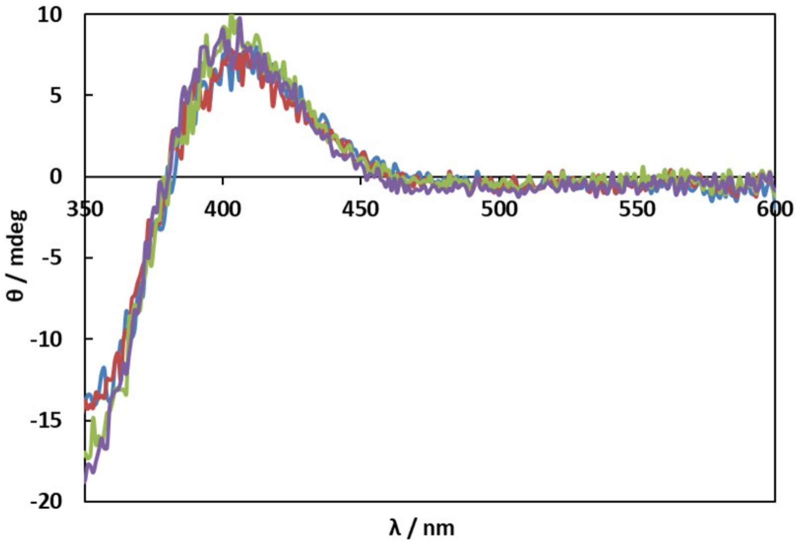


**Fig. S1** CD spectra of solutions containing **1** (2.00 mM) and 4.00 mM (blue), 8.00 mM (red), 12.00 mM (green) and 16.00mM (purple) of L-Ala at pH 5.0.

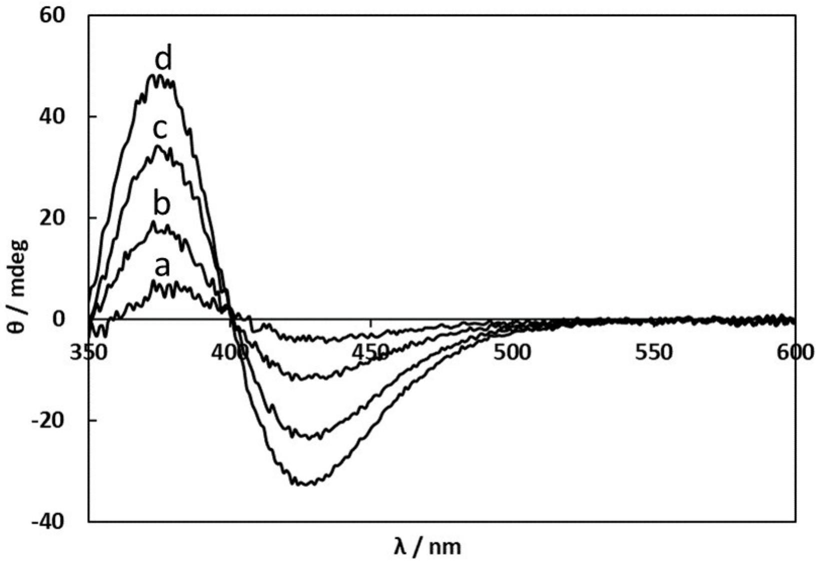


**Figs. S2** CD spectra of solutions containing **1** (2.00 mM) and 4.00 mM (a), 8.00 mM (b), 12.00 mM (c) and 16.00mM (d) of L-Ala at pH 11.0.

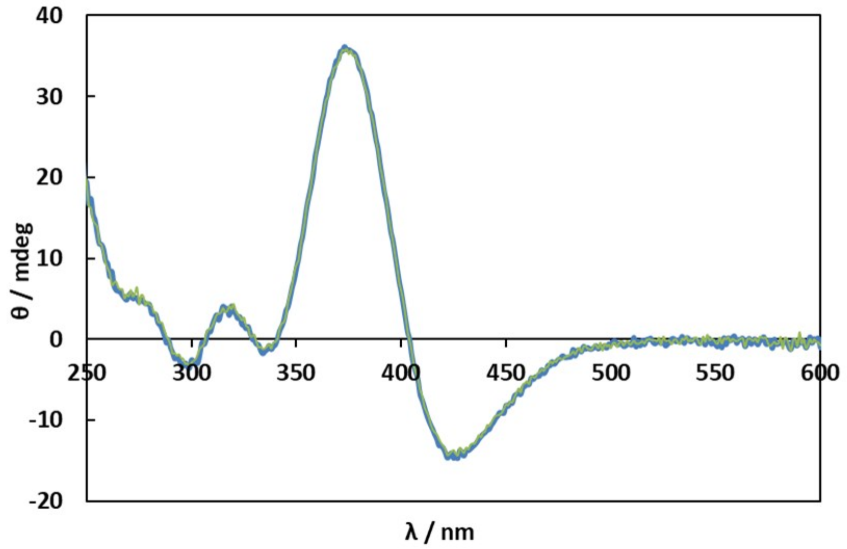


**Fig. S3** CD spectra of solutions containing **1** (0.200 mM) and L-Met (0.400 mM) at pH 2.03 (blue) and pH 11.01 (green).

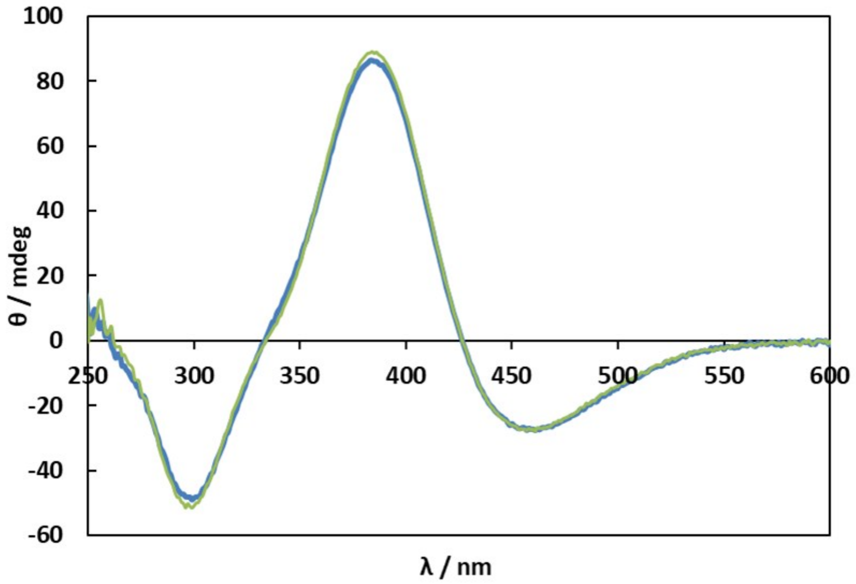


**Fig. S4** CD spectra of solutions containing **1** (0.200 mM) and L-Pen (0.400 mM) at pH 2.03 (blue) and pH 11.02 (green).

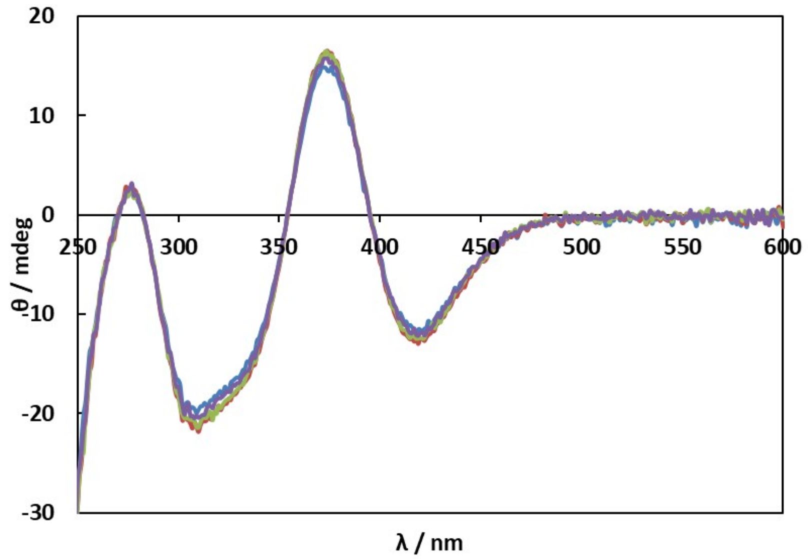


**Fig. S5** CD spectra of solutions containing **1** (0.200 mM) and 0.400 mM (blue), 0.800 mM (red), 1.20 mM (green) and 1.60mM (purple) of *S*-Me-L-Cys at pH 2.0.

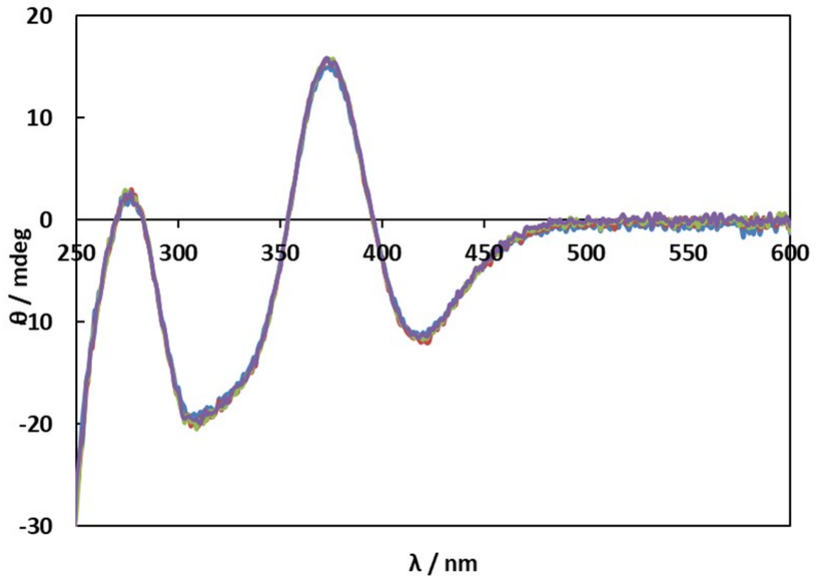


**Fig. S6** CD spectra of solutions containing **1** (0.200 mM) and 0.400 mM (blue), 0.800 mM (red), 1.20 mM (green) and 1.60mM (purple) of *S*-Me-L-Cys at pH 11.0.

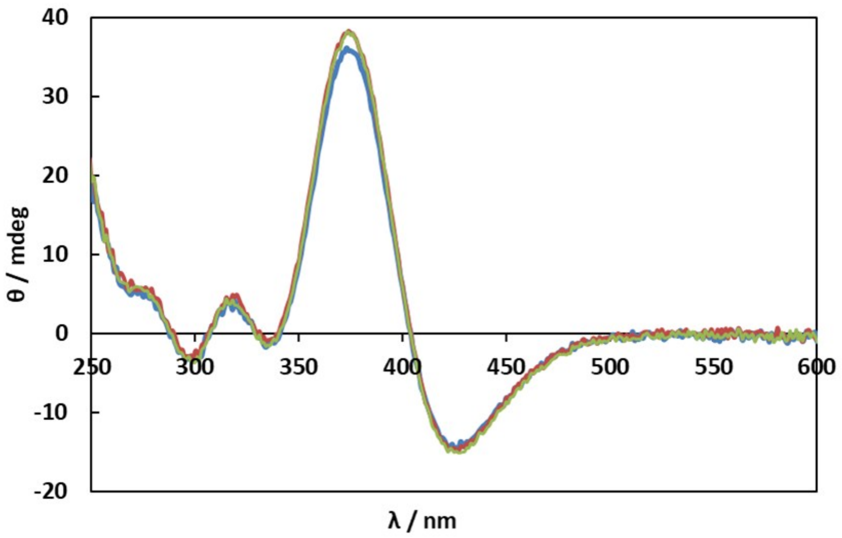


**Fig. S7** CD spectra of solutions containing **1** (0.200 mM) and 0.400 mM (blue), 0.800 mM (red) and 1.20 mM (green) of L-Met at pH 2.0.

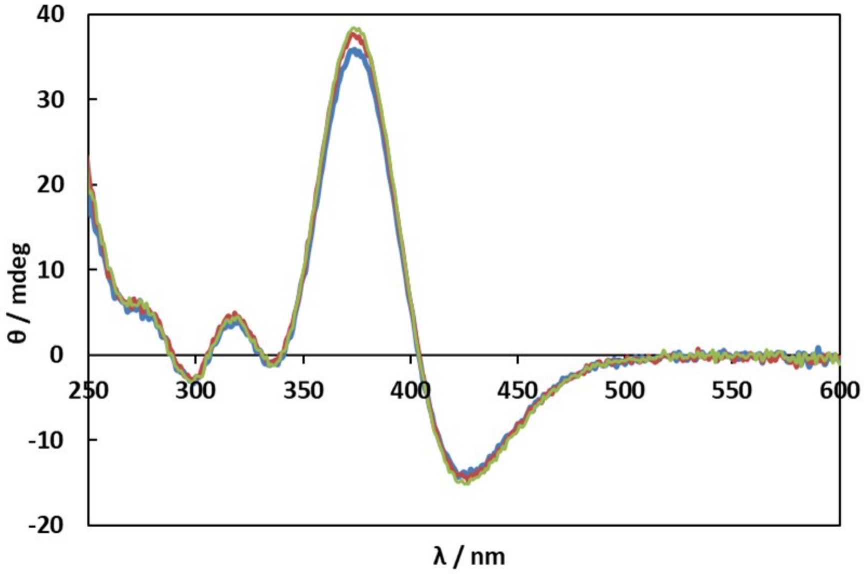


**Fig. S8** CD spectra of solutions containing **1** (0.200 mM) and 0.400 mM (blue), 0.800 mM (red) and 1.20 mM (green) of L-Met at pH 11.0.

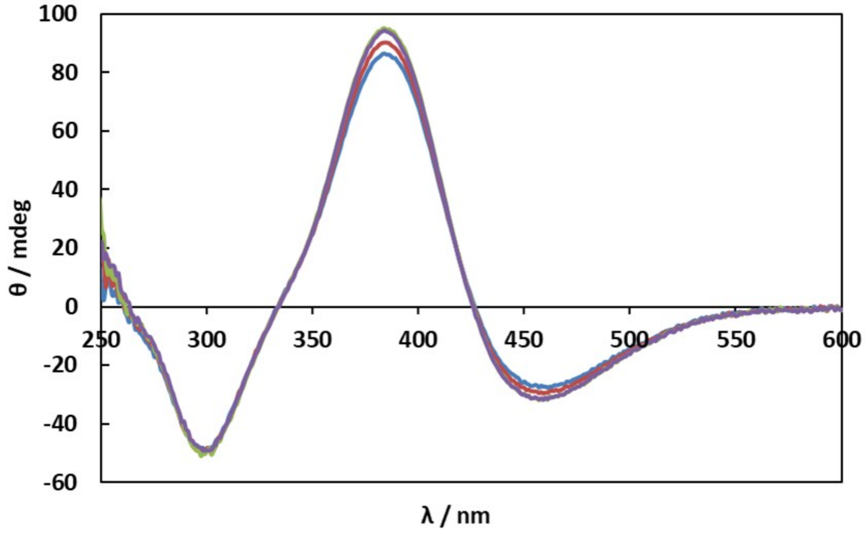


**Fig. S9** CD spectra of solutions containing **1** (0.200 mM) and 0.400 mM (blue), 0.800 mM (red), 1.20 mM (green) and 1.60mM (purple) of L-Pen at pH 2.0.

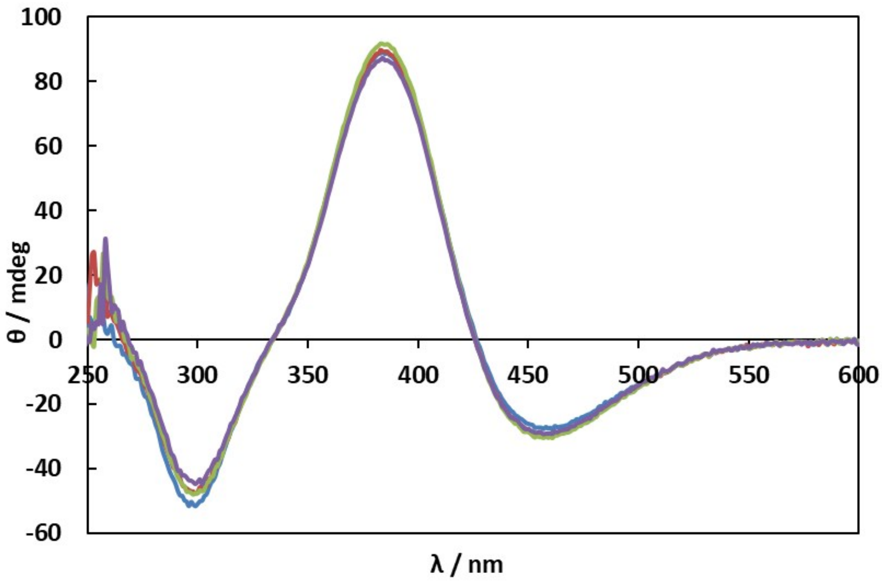


**Fig. S10** CD spectra of solutions containing **1** (0.200 mM) and 0.400 mM (blue), 0.800 mM (red), 1.20 mM (green) and 1.60mM (purple) of L-Pen at pH 11.0.

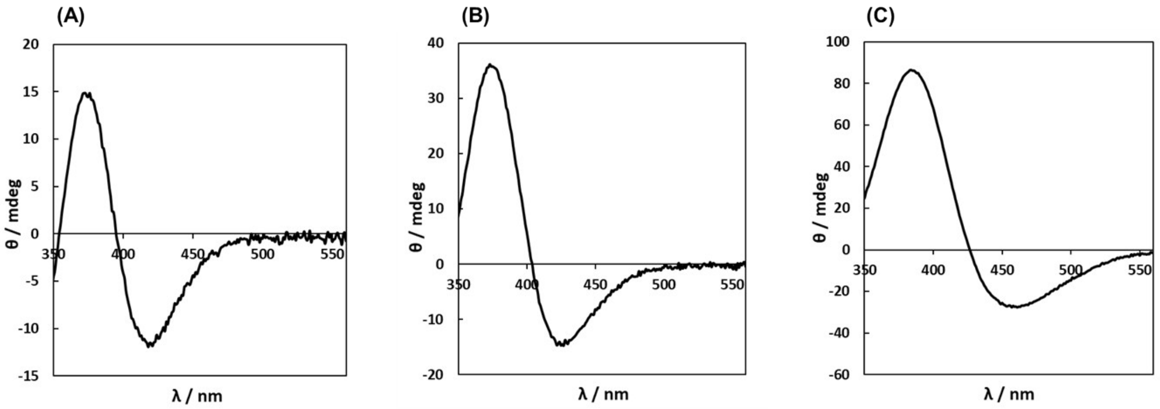


**Fig. S11** CD spectra in the visible region of solutions containing **1** (0.200 mM) and 0.400 mM of *S*-Me-L-Cys (A), L-Met (B) and L-Pen (C) at pH 2.0.

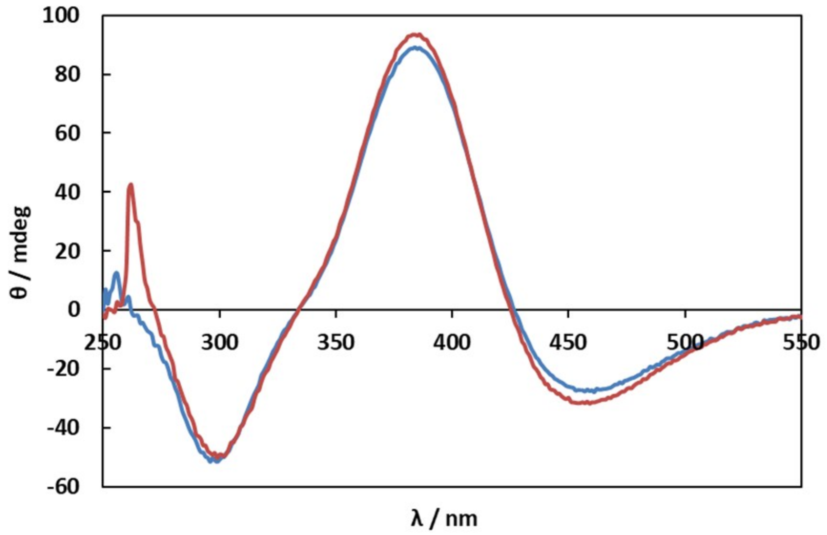


**Fig. S12** CD spectra of solutions containing **1** (0.200 mM) and 0.400 mM (blue) and 40 mM (red) of L-Pen at pH 7.2.

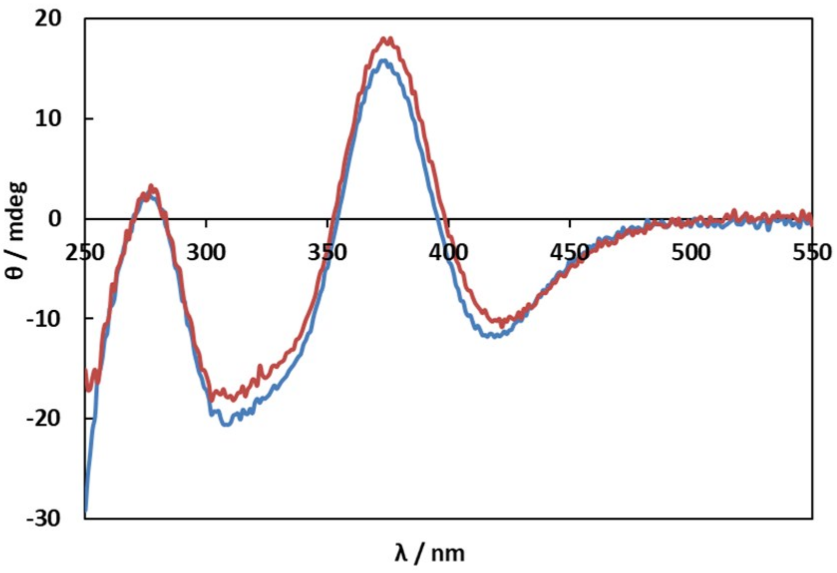


**Fig. S13** CD spectra of solutions containing **1** (0.200 mM) and 0.400 mM (blue) and 32 mM (red) of *S*-Me-L-Cys at pH 7.2.

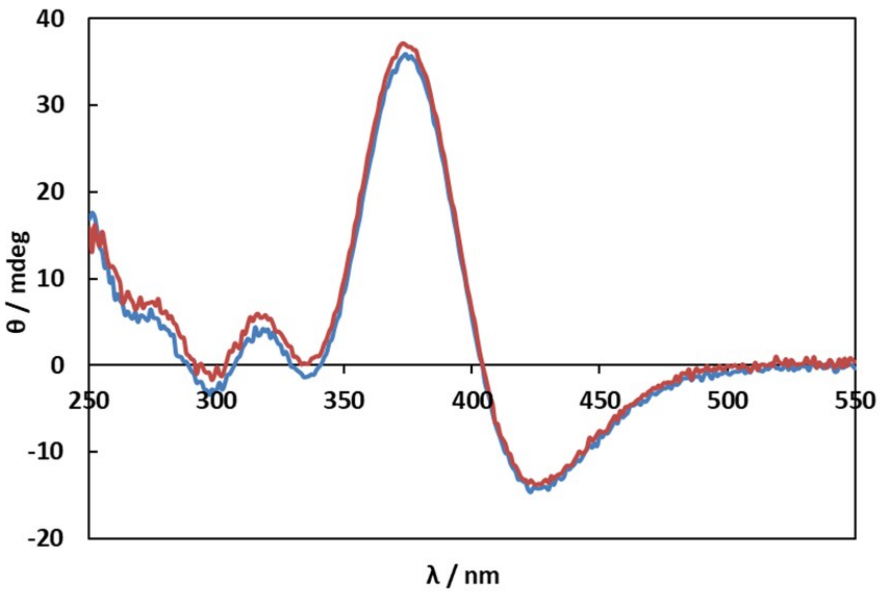


**Fig. S14** CD spectra of solutions containing **1** (0.200 mM) and 0.400 mM (blue) and 60 mM (red) of L-Met at pH 7.2.
